# Syphilis

**Published:** 1868-01-15

**Authors:** W. F. Peck

**Affiliations:** Davenport, Iowa, late House Surgeon, Bellevue and Blackwells Island Hospitals, New York


CHICAGO MEDICAL JOURNAL.

*Vol. XXV.—JANUARY* 15, 1868.—*No.* 2.

### SYPHILIS.

*A Report made to the Iowa State Medical Society, by Dr. W. F. Peck, of Davenport, Iowa, late House Surgeon, Bellevue and Blackwells Island Hospitals, New York.*

HAVING been assigned by the Society to report on the surgical diseases of the organs of generation, I purpose considering, at some length, the disease which, above all others, most interests the physician and surgeon. Inasmuch as its consideration, in the strict surgical sense, would be, from necessity, incomplete, I design making use of a latitude best calculated to carry out the import and interest of the present paper, as well as elicit opinions, hitherto unexpressed, in this organization. In analyzing the subject of Syphilis, the time is not to be consumed in making researches into its historic origin and early insinuation into human society.*

What benefit does it confer on civilization to know whether Moses and the Israelites suffered from specific contagion, or whether the whites of America and the modern population of

* At the meeting, in 1866, I made a report considering, at length, the history of the disease.

the Old World received the disease from the American Indians, and by way of Kamtschatka?

The professional world has, with each succeeding century, advanced and retrograded with such strict parallel, that until within the second quarter of the 19th century, our apparent permanent improvement, in developing practical information relative to the diagnosis and treatment of Syphilis, has resulted in comparatively little benefit to those whose misfortune it is, and has been, to be tinctured and saturated with the most treacherous and destructive of poisons—*Syphilis.*

Recognizing, as has been done for many years, the division of specific ulcerative diseases of the generative organs into two classes—viz., Syphilis, by which I desire to be understood as meaning a local sore on the penis, or elsewhere, produced generally by sexual intercourse, and, at the same time, currently disseminating the specific poison throughout the entire system, by means of the circulating fluid, and sooner or later manifesting itself in the cutaneous-glandular membranous, ligamentous, and osseous systems, and syphilides, mites, or chancroids, or a local ulcerative sore, the ravages of which are purely local only when the system secondarily suffers from the local ulcerative irritation. I design arranging the subject into these two general heads, and regard them as entirely separate and distinct from each other, yet, both being specific, and ordinarily received under similar circumstances, and in the same way. Syphilis is generally conceded to be communicated at the time of intercourse, and many have, and still continue to assert, teach, and practice, that the poison can only be communicated to a second person through an abrasion or ulceration of the tissues.

While I believe that the disease is often—I may say usually—communicated in this way, I also believe that it is often transferred from one person to another without even breaking continuity of tissue.

In an individual bearing a chancre, there is, at the same time, the latent poison circulating in the blood and secretions of the body, all of which are taken from the blood immediately before their consummated preparation is known by a distinctive name.

It is by no means necessary that the individual should have a chancre at the time of communicating the poison.

I believe that a person who has never had the disease can receive it from a person who may have it, by actual contact in almost any form—through the semen—contact of the hands—contact of the body, as in sleeping—and, in fact, any position of the body which will permit a transfer of communicable poisons. If the poison of Syphilis is received into the blood, and, like the inexplicable leaven of the Pharisees, is disseminated throughout the system, why should not the secreted fluids, which are a separated part of the blood itself, contain some of the poison with which it has been associated in its earlier existence. No one at the present day doubts the hereditary history of Syphilis.

If the poison contained in the germinating fluid of the male be communicated to the female through the segmentation of the ovum, made so by the spermatozoa of the semen, why can not the semen, which remains for an indefinite length of time, in many cases, in contact with the absorbent vessels and cells of the vagina, be absorbed, or the poisonous leaven of the semen be absorbed, and thereby be made to come in contact with every living cell in the human economy.

It is not necessary at this time to attenuate on the absorptive capabilities of the skin and mucous membrane. Admitting that agents of atmospherical and chemical nature are admitted through these several tissues, why, I ask, in the same connection, can not the unanalyzed and mysterious poison of Syphilis be introduced or admitted into the system through the same avenues?

It may be asked, can a male who has not Syphilis receive it from a female who has the disease in its secondary form, without receiving it through tissue broken by the specific poison? There is always a natural secretion in the uterus and vagina, and generally there is in woman what may be termed a hypersecretion, from the cells there located. If the blood from which the fluid is separated contains the poison, as before remarked, why should not the Glandulæ Tysmii absorb or take from the fluid the poison, without abrading the tissue? And, just at this juncture, I desire to state that I believe that a chancre can be produced by a person who only has the disease in the second and last form.

In doing so, the tissue cells are worked excessively by the irritating or poisonous fluid, and in thus laboring, the walls may be broken in some of them, and the adjoining cells, in exerting themselves in the effort to repair, in succession break down, and the result is, an ulcer or chancre, specific in character. In the summer of 1866, a gentleman applied to me for treatment of ulcerative disease of the glans-penis and prepuce. I found several ulcers on the different parts of the organ mentioned, and accompanying them there was badly strictured phymosis. The phymosis was so complete, that I could not obtain a good view of the multiple ulcerations.

The diseases were all removed and remedied by circumcision. No difficulty intervened to hinder an uninterrupted recovery.

About two months subsequent to the operation, a lichenous eruption appeared on the face, arms, and back. I immediately placed the patient on specific treatment, when the local evidence disappeared; but within twelve weeks, I was called to treat the wife of the gentleman, whose age was 30. She had never had offspring. She first complained of sore throat, and soon after, the face was the seat of a specific acneated eruption.

The system became exhausted, and, as a consequence, a state of morbid nervousness was prominent. Ulceration appeared in the nose, and soon after some fifteen ulcers appeared on the inferior extremities. Under suitable management, the signs and symptoms subsided, and she is now apparently well. No sore of any kind ever existed on the genital organs. Upon minute inquiry, I found that no preventative was employed by either party to avoid pregnancy.

Intercourse was rather often, and the seminal fluid remained within the vaginal cavity. She had more or less leucorrhœa, caused, I believe, by the irritating presence of the male fluid. Both patients are still under my professional care.

CASE II.—In 1863, Mr. B—— had an ulceration on the glans-penis, which occurred some ten days after intercourse with a woman whom he believed to be healthy in every respect. He was treated a short time, and discharged cured. In 1865, he applied to me for advice in relation to his wife's condition, which was as follows. She had had an eruption, I should think from the description given, of psoriasis, which lasted for several months, when neuralgia became an almost constant trouble. She also had iritis and ulceration of the throat, followed in a few months by periostitis of both tibial membranes. She had never had an ulceration of any kind on the organs of generation. I requested an examination of the husband, and found on the left tibia a node which had given him a good deal of nightly trouble, but thinking it was benign rheumatism, he used simple remedies, all of which afforded him no relief.

I have treated both husband and wife, and they are now apparently free from specific disease. They have one child, five years of age, and perfectly healthy. Upon inquiry, I found that no precaution is, or ever has been used to guard against pregnancy. Intercourse averages once in five days, and the seminal fluid remains unremoved from the vagina. From the above cases, together with the vast amount of accumulative testimony on record, I am well satisfied that an abrasion of the surface is not necessary to transfer the poison of Syphilis from a first to a second party. All of the effects of *Aconite* and *Belladonna* can be produced by causing them to come in contact with the absorbent vessels, and still more authentic record has proved, that although a long time might elapse, yet the virus would eventually act on a healthy skin, after having, of course, completely sodden the epithelial covering.

Babbington, the commentator of Hunter's written opinions says, "The character of the primitive venereal affection is *essentially* an induration *passing* afterwards into an ulceration." Does this argue less than absorption first, with breaking down of cell tissue after the system at large has been contaminated? Hunter himself entertained this opinion, but owing to a want of distinctive practical proof, he refused to introduce his belief into the form of a general histological law. Diday gives a case in his memoirs, where a syphilitic infant was introduced into a small village to be nursed by a wet-nurse who received the disease, and afterwards was the means of propagating it to a considerable number of the town population. Dr. Bargioni, an Italian physician, who was so sceptical in his belief in science, could not receive any other opinion than that entertained and taught by Ricord and Europe, years ago, and, willing to give practical demonstration of his belief, he suffered the blood to be taken from a woman who had the disease in its secondary form, and inoculated into his own system. In four days all signs of the inoculation were gone. After a few days, he noticed an itching, and, on looking at the arm, there was observed a round papule of a red color, but no induration around it. The papule gradually increased, and in eight days was of some size, and covered with a silvery scale. Eleven days after its first appearance, two glands in the axilla became enlarged. On the sixteenth day, these glands were larger, and upon removing the papule, a small quantity of serosity was found beneath, but no induration around it. On the eighteenth day, there was an ulcerated surface, with a crust on it, and a certain amount of hardness at its borders. On the twenty-third day, it was larger and harder, as were also the glands. A month after the first appearance of the papule, he had nocturnal pains in the head, and observed some enlargement of the cervical glands. A week after this, a roseolous rash appeared on the body and spread all over him, leaving no doubt of its nature. In another week, the sore was not disposed to heal, and the glands were larger. The rash then became copper-colored. Remedies were then given, and the sore commenced to heal.

If medical men are averse to these opinions, why are they so particular in making inquiry after pure and healthy vaccine matter. I might go on multiplying evidence, but I think already sufficient has been adduced to confirm what I set out to prove.

Chancroids — or — in order to consider the specific malady, Mild Syphilis — I will term it Syphilides Mites, although I scientifically consider them entirely separate and distinct diseases. Chancroids are generally received at the time of intercourse, and the ravages are always local until the irritation has provoked an exhausted system, which may induce the attendant to regard the disease as Syphilis. There are no stated or constant descriptive symptoms which can be relied upon at all times. Those careful writers and observers who see in chancres invariably the circular, even-cut, half-split-pea excavation, with circumscribed indurated base, see in the chancroidal ulcer an irregular, ragged, superficial ulceration, with a disposition to spread or extend its area of surface rapidly, and no tendency to respect geometrical angles or circles. The fluid — the result of all disintegration — is of a grayish, yellowish color, and usually abundant in quantity, and wherever it may chance to be deposited it manifests the same creative tendency, and where in Syphilis the ordinary disposition is to form but one ulcer, in chancroidal disease the tendency is always to exhibit multiple characters.

If the system of a patient bearing a chancre, or multiple chancre as sometimes happens, becomes depraved and exhausted, the same disposition to wander, ravage and destroy may be seen; but these conditions are far less frequent than they are in specific disease denominated chancroids. The eruption, iritis, falling of the hair, pharyngitis, ulceration of the throat, including vocal chords, mucous and osseous ulceration of the nose, periostitis, and ulceration of soft tissues in all parts of the body, and, lastly, caries and necrosis of the bones are almost always confined to, and symptoms of, Syphilis proper.

Hereditary Syphilis is a disease too little studied, and, consequently, too little understood by the medical profession.

How far in a pregnant woman the structures connected, with the nourishment of the fœtus in utero may suffer from the effects of Syphilis has not yet been determined with scientific accuracy.

The still-births and abortions by women affected by this disease are too common to receive extended notice. But in these instances the cause is in the fœtus itself; it is diseased, dies, and is thrown off. But miscarriages are by no means infrequent in tainted women, even when the fœtus itself shows no signs of disease. In such cases it has been satisfactorily shown that the placenta has been the seat of specific deposit. Children who are born with Syphilis are recognized by their impoverished look and morbid condition of the tegumentary system. When the disease is not prominent at birth a roseolous eruption, sometimes lichenous, appears, accompanied by ulceration in the mouth, and very often snuffles, together with condylomata, and sometimes the eyes exhibit a marked evidence of the systemic taint. Under treatment, and often without, these cases get well within a year, and the wise parties are satisfied that the disease has been cured. But sooner or later the poison re-presents itself, and the patient shows a puny, ill-developed constitution, and is constantly troubled with some disorder of the organization.

Interstitial keralitis and deafness and, later, organic renal disease are prominent. What surgeon can recognize the greatest of human ills in the infant or youth and not sympathize sadly as he reads the prophetic edict in the countenance of the innocent. "The sins of the fathers shall be visited upon the children, even unto the third and fourth generation."

With an understanding knowledge of Syphilis, the most important of all questions is that of treatment.

Treatment.—I treat all ulcerations of the penis specific in character with the most active escharotics. Fuming *Nitric*

*acid* and *Bromine* are favorite medicines, and when properly used supply the demand better than any other agents that I have ever employed.

In chancroids I cauterize the ulcer to *cure* the disease, and also to prevent its being communicated to a second person.

In chancres I cauterize the ulcer to prevent the disease being communicated to a second party, not because I entertain an idea that the progress of the disease in the person bearing the chancre can be arrested by any kind of local treatment.

Whenever the chancre assumes a serpiginous or gangrenous ulceration, I apply precisely the same course adopted in treating chancroids. In both diseases, when the specific character of the ulcers is destroyed, I apply to the surface an ointment composed of equal parts of *Bals. Peru* and *Spermaceti Ointment*, which is immediately covered with a fine quality of oakum, better calculated than any local absorbent to facilitate the healing process of granulating ulcers. Lint, and all other applications of a similar nature, are far inferior in my practice to oakum.

Daily applications of the ointment and oakum will generally suffice. But should the patient be convenient, twice a day, immediately after the cauterizing, will be better. This method of treatment in chancroids is peculiarly adapted. Should the ulcers not heal readily, and should the granulations manifest a sluggish disposition, a daily syringing with the following will usually modify the action of the ulcer and assist materially in establishing a healthy process:

℞

| | |
|---|---|
| Glycerin, - - - - | ℥i |
| Creosote, - - - - - | ʒi |
| Tr. Myrrh, - - - - | ℥i M. |

Put two drachms in two ounces of water, and syringe the parts thoroughly immediately before dressing.

While this preparation furnishes ample disinfecting properties, it also operates admirably in multiplying healthy cells.

In employing the local plan sketched, the wants or necessities of the system at large are by no means to be overlooked or neglected. Beef and mutton in substance and fluid extracts are to be especially administered. Stale bread, milk and potatoes are also to be used daily. He who allows an abstemious diet to enter into his treatment will suffer a want of corresponding success. Daily baths containing a small amount of *Sulphuric acid*, are both grateful and valuable. Whenever the granulations manifest a very active disposition, use a little *Nitrate of silver*, as often as indications require, immediately within the line of circumference, until new integument forms the future cicatrix. I am not of the opinion that *Calomel*, as a local application, in chancres, is of any more value as a healing remedy than *Bismuth* or *Magnesia*. Almost anything that will absorb and remove the irritable secretion will aid the formation of new tissue. In my hands *Tannin* acts admirably as a dry remedy.

The time for commencing internal treatment in Syphilis is agreed upon by all observers to be when the disease is recognized. Notwithstanding my practical and observatorial experience in this disease, I am very often, and I might say *too* often, at a loss to discriminate between Chancroids and Syphilis, as presented on the penis. And, rather than inflict upon my patient a course of *Mercury* and other depletant remedies unnecessarily, I wait until the skin osseous or mucous tissues give me information positive as to the location of the disease in the circulating fluid. I am aware that this course of management will be criticized. But calm, reflective consideration should be used in analyzing the disease, together with its development, and it ought to appear evident that it is better to wait only a little longer and be certain that the disease exists, than to act on false or uncertain supposition, and thereby provoke incalculable injury.

I do not desire to be understood as saying that the effects or results of medicine used in treating Syphilis are, or can be, as injurious as the poison itself. In my opinion, no other poison or disease can be compared to Syphilis in its ravages and devastating process.

Legitimately, Syphilis has but two stages, although writers and commentators arrange it under three stages, viz.:

The first to include the disease from the time the poison is received up to the time it shows systemic development.

The second and last — from the time it shows systemic development up to the time it terminates either in death or recovery.

When I am perfectly satisfied that the penis augurs Syphilis, I immediately commence internal treatment.

Mercurial ointment, with occasional small doses of *Calomel*, is, and has been, in my hands, by far the best remedy. It does not derange the digestive organs and thereby deprive the surgeon of an all-important adjuvant in treatment.

The effects of the medicine are almost as quickly produced as when remedies are given internally. At the same time, *Mercury* can best be controlled in form of administration. I direct it to be used in the axilla, groin, and popliteal space alternately, on opposite sides, and as often as the condition of the patient demands it.

When it is not convenient to use the ointment, I use *Proto-iodide of mercury* pills. But when the remedy is to be continued for a long time, I use the *Corrosive chloride of mercury*. In the last part of the second stage, I find no remedy that will supply the place of *Iodide of potassium.*

I give, however, much larger doses than is usually recommended. Often the *Potassium* will so accelerate the action of the glandular system as to bring or send out the eruption more profusely. When it does, disregard the solicitations of the patient, and the eruption will disappear much sooner under the *Potassium* than it will under the *Mercury.* Never fail to keep a watchful eye on the digestive system—good food and plenty of it must always be insisted upon. A healthy or active digestive system will assist very materially in neutralizing and throwing off the poison.

I have tried vapor-baths, but all plans of treatment are secondary in my hands to that described and advised. Whenever any of the glands give evidence of the formation of matter, let it out. Syphilitic glands rarely suppurate. Sympathetic bubos are common. Sometimes *Iodine* has a discussive influence, but less frequently than is believed. A saturated solution of *Nitrate of silver* will usually remove satches or ulcerations of the mucous membrane.

I have no confidence in the plan of Boeck. In 1862, I witnessed, in Blackwells Island Hospital, a series of unsuccessful experiments with syphilization, by Dr. J. E. Taylor. I never grant a final discharge to my patient in a less time than one year, and sometimes a much longer period is necessary to cure the disease.

I hope, from time to time, to review this interesting subject in the deliberations of this organization.

